# Burrowing into the legal system: using interview methods while respecting the theoretical basis of systems theory

**DOI:** 10.3389/fsoc.2026.1854990

**Published:** 2026-06-23

**Authors:** Emer Hunt, Gerardine Doyle

**Affiliations:** 1 School of Law, University College Dublin, Dublin, Ireland; 2 Michael Smurfit Graduate Business School, University College Dublin, Dublin, Ireland

**Keywords:** interview methodology, Luhmann, social action, systems theory, tax avoidance, morality, state aid

## Abstract

There is a difficulty reconciling the anti-humanistic approach of systems theory (as developed by Niklas Luhmann) with methodologies centering the individual. Society is viewed as a constellation of systems – politics, law, economy, media – and not the agglomeration of billions of individuals. This fore fronting of systems, and the unknowability of the cognitive processes of individuals, makes empirical operationalization of systems theory difficult. Can an interview method ever be accepted as a robust empirical operationalization of a theory which views society as a collection of intertwined and self-reinforcing systems, recursively communicating according to their own internalized contingencies? This paper attempts a theoretical justification for a methodology where individuals (researcher and interviewees) observe a system from which, theoretically, the human agent is excised and the focus is only on the construction of systemic possibilities. Reconciling theory and method is an important step in recognizing the possibilities of systems theory as a way of examining society.

## 1 Introduction

This paper explores whether a social theory (Luhmann, 1995b) that emphasizes social action as a series of systemic communications, rather than merely the aggregation of individual agency (Nobles and Schiff, 2020), can be combined with a methodology involving interviews with elite individuals. The empirical difficulties that arise from a tension between a sociology of social action and a sociology of social structure have long been recognized (Dawe, 1970). This paper addresses the methodological tension arising from these two strands by situating interviews with individuals within the constraints and influences of social structures through a second-order observation (Christis, 2001; Geimer, 2015). Elite individuals — such as policymakers, corporate executives, and professional advisers — are often agents in regulatory and legal processes. Yet their perspectives cannot be isolated from the broader structures of meaning, possibilities, and institutional practice in which they operate.

Bridging this gap between systems theory and social action method is tricky. The paradox of systems theory is that, while it is a theory of systemically bounded communication, operationalizing this theory relies upon some method to observe systemic communication. The research accepts legal rulings as communications of the legal system (Luhmann, 2004). Knowledgeable individuals assess these communications, probing how the legal system was prodded into action, considering how one (and not another) systemic communication was issued, and observing how discussions within the political and economic systems might be translated into possibilities of the legal system (Harste and Febbrajo, 2016). In doing so, the research examines how meanings are constructed through communicative practices, including latencies of justification, strategic framing as a matter of fairness or equity, and the invocation of morality within the legal system. These latencies may not be evident in the communication itself, as that is clothed in the autopoietic possibilities of the systemic (legal) code.

Yet, at the same time as this second-order observation of the legal system is undertaken, there is fundamental theoretical acceptance that the interviewees are not merely expressing a personal preference, a desired agency. This theoretical lens moves attention away from the potent individual, not seeking to ascribe motivation or political bias to their unknowable cognitive processes (Luhmann, 1995b) but instead addresses the ‘how’ of a system maintaining its boundaries while internalizing external influences. Interviews are a second order construction of the meaning of a legal ruling, a reflection on and, indeed, reproduction of the social and institutional logic that structures regulatory enforcement and legal interpretation. It is, therefore, a knowingly slantways observation to unpick the contingency and latencies of a specific finding of illegality by the legal system.

The remainder of this paper explores this problem and is organized as follows. In Section 2 (The empirical context: a tax law dispute involving Ireland, Apple and the European Commission), the background is to the legal dispute is outlined. Section 3 (Theoretical basis of the research: social systems), explains the theoretical basis of systems theory is, including the device of second-order observation to clarify latencies and blind spots. Section 4 (Deciphering the empirical gap) explores the sparse and disjointed empirical literature on systems theory. Section 5 (Materials and methods) explains the choice of interviewees and how the interviews were analyzed. Section 6 (Discussion: coherence and limitations of empirical application of systems theory) assesses the success of operationalizing systems theory in an empirical context.

## 2 The empirical context: a tax law dispute involving Ireland, Apple and the European Commission

The empirical context is a legal dispute involving Ireland, Apple and the European Commission where, in 2013, the EU began to investigate Ireland’s tax treatment of Apple contained in two tax rulings dating from 1991 and 2007 (Mason and Daly, 2024). This could be seen as the legal system being prodded to respond to a political noise. In 2013 Apple appeared before the US Senate (2013) to be questioned by politicians about its tax practices, characterized by some as questionable and dubious and by others as the legal minimization of Apple’s tax burden. Tim Cook, chief executive officer of Apple, used compliance with the legal system as a defense to robust questioning by politicians: “We not only comply with the laws, but we comply with the spirit of the laws. We do not depend on tax gimmicks” (US Senate, 2013, p. 37).

The legal system is not static, but autopoietically dynamic. The EU legal system may well have taken note of Tim Cook’s characterization of how Apple was recruited by Ireland:

…so as a part of recruiting us, the Irish Government did give us a tax incentive agreement to enter there, and since then we have built up a sizable operation there, nearly 4,000 people.

A tax incentive agreement, seen by Tim Cook as a sweetener to locate to Ireland, could resonate in the legal system as a possible illegal state aid. Tim Cook’s appearance before the US Senate took place in May 2013 and, by June 2013, the European Commission had launched an investigation into that tax incentive agreement.

This legal investigation by the EU itself reverberated in the political system as the U.S. Department of the Treasury (2016a) threatened to “consider potential responses should the Commission continue its present course” (U.S. Department of the Treasury, 2016b), with the size of the monetary penalty leading to accusations of the opening of a trade war between the EU and the USA (The Economist, 2016; Chee and Halpin, 2016; Treanor and Monaghan, 2016). Thus both legal and political systems took notice of the tax practices of Ireland and Apple.

The legal argument pivoted on whether Ireland had given a ‘tax incentive agreement’ specifically to Apple in 1991 and, again, in 1997. Preferring one taxpayer over other taxpayers could fall foul of the state aid rules prohibiting an EU member state providing preferential treatment to industries, sectors or particular taxpayers. In the Ireland-Apple case, the state aid was argued to be the selection by Ireland of Apple for favorable tax treatment (Navarro, 2024). The investigation by the European Commission, which had begun in 2013, concluded in 2016 with the Commission ordering Ireland to recover €14 billion in tax underpaid by Apple (Mason and Daly, 2024). After several legal twists and turns, this order was upheld in September 2024 — and Ireland’s actions categorised as prohibited state aid — by the apex court of the EU, the Court of Justice. Paradoxically, although Ireland lost the state aid legal case, Apple was obliged under law to pay the amount of tax which it had underpaid and, therefore, Ireland recovered €14 billion in additional corporation tax, so adding to Ireland’s already healthy tax revenues (Mason and Daly, 2024).

This long-running triangular dispute between Ireland, Apple and the EU could be studied as a legal, economic or political event, resonating in all these domains – or systems – and being assimilated into the particular logic and practice of each system. Each system is functionally separate: the legal system viewed Ireland’s tax treatment as illegal; Apple paid €14 billion in tax. The political system sees Apple’s location in Ireland as a result of tax competition by states and tax avoidance by multinational enterprises and it continues to grapple with these issues (Haase and Kofler, 2023).

## 3 Theoretical basis of the research: social systems

Drawing on systems theory, as developed by Luhmann (1995b, 2004), and associated, particularly within the context of the legal system, with Teubner (1987), the EU’s findings of illegal state aid by Ireland to Apple are posited to be communications of the legal system. Luhmann proposed that society is composed of various self-reproducing functional social systems, each with its own distinct logic and mode of operation. These systems include the economy, law, politics, religion and science. According to Luhmann, social systems operate through communications and are autopoietic, meaning they produce and reproduce themselves, building communications based on previous communications, influenced (but not dictated to) by the communications of other systems. Luhmann’s theory of the differentiation of modern society into various functional systems, in seeing society as composed of systems and environment, was heavily influenced by the US sociologist, Talcott Parsons (Vanderstraeten, 2021).

There are several interrelated premises necessary to draw out the epistemological basis of the empirical work. These are explained, together with a discussion of how the theoretical basis informed the interviews.

The first premise is that the epistemic subject is the system itself. Very often, the political system is viewed as the superior system, the driver or the representative authority for all other systems (King and Thornhill, 2003). In this research, the epistemic subject is the legal system. This is a particularly bounded system, with clear communications of illegality (2016), legality (2020) and, in a reversal, illegality (2024). The focus of the empirical work is, therefore, the various legal communications which issued from the EU.

What this means, in empirical work, was that there was no emphasis on personalities, no focus on individuals driving the action against Ireland and Apple, or resisting the EU action through legal argumentation, or political lobbying. There were no questions as to “who did what”, but only questions, from an internal point of view, as to how the legal system formulated a sophisticated (and much disputed) legal argument. This internal analysis depends upon deciphering the autopoietic operation of the legal system, perhaps through a doctrinal analysis. Legal doctrine is “only an attempt to solve ‘similar’ cases consistently” (Luhmann, 2004, p. 258). Once irritated by the environment, the legal system responds through its internal formulation of the problem and solutions are “constructions within the reach of law” (Luhmann, 2004, p. 28). The state aid verdict of illegality was, therefore, examined to determine how it compared with similar cases and how it was a viable construction under legal doctrine.

This could be the end of placing the legal system as the epistemic subject of the research. However, the straitjacket of communicating in a binary code, together with a recognition that the autopoietic legal system communicates in an ostensibly dry, technical form, far removed from an expression of disapproval and bleached of values, means that an external observation of the legal system can also be carried out to uncover latencies of meaning. This is not a move to personalities and individual cognition. Instead the system is examined, using individuals as observers, to understand the process of systemic communication: to identify what communications from the political system, or from the media system resonate within the legal system; to tease out the problems to which the enforcement action by the European Commission was a solution; to observe the distinctions made by the legal system. This construed the system as obeying its constraints but also shaping itself to tackle tax avoidance by multinational enterprises, a lack of business ethics, the lack of political will to move tax from the nation state to a wider geography, and the tax competition by states seeking to attract multinational enterprises with low tax rates. Accepting the contingent operation of the legal system in any particular circumstance (Luhmann, 1995a) enables the hindsight bias of doctrinal research (van Gestel and Micklitz, 2014) to be overcome. The state aid action taken by the European Commission against Ireland and Apple was not pre-ordained or inexorable: it might never have been taken (indeed the “tax incentive agreement” dated from 1991) (Luhmann, 2012), or it might have been held to be legal (as the General Court of the EU held in 2020) (Parada, 2021). The sheer unlikeliness of the legal system communicating in a particular way, at a particular moment in time is, therefore, a study of systemic possibilities, not as “aggregates of preferred motives for action” (Luhmann, 2012, p. 15).

Secondly, observation requires the “making a distinction to indicate one side and not the other” (Luhmann, 1993, p. 774). Using an interview method, these observations are constructed through second-order observation, a process whereby the researcher observes the interviewees who are themselves observing the communications of the legal system. Second-order observation, as the perception of what others say or do not say (Luhmann, 2013), is important in the analysis of the interview data and thus becomes a methodological signpost in the construction of social meaning. Luhmann writes that: “Real is what is practiced as a distinction, what is taken apart by it, what is made visible and invisible by it: the world” (Luhmann and Rasch, 2002, p. 65). This is a very modern realization: the interpretation is the reality, and each reality is based on the observing distinctions through which an observer views reality.

Therefore, systemic communications are observable, but only as a construction of the observer. This observation is fragmented, which leads to the realization that there is “no access to a commonly assumed objective world” (Rasch, 2000, p. 33) or there is no social reality “out there” (Teubner, 1989, p. 730). This is the epistemological departure of systems theory; a new way of expressing the construction of social realities. It is allied to an understanding that the autopoietic conceptualization of law is antirealist and anti-individualist.

An example may help here. When conducting interviews, the first distinction was between the system and its environment, which meant that the epistemic subject of the interview was how the legal system communicated on a particular situation – the tax rulings given to Apple by Ireland. Interviewees recognized that distinction but also observed the legal system as a problem-solver, responsive to a great many social and political problems and observing the distinction between fair and unfair, moral or immoral, to arrive at a ‘fair’ judgment. There was a degree of fuzziness in ascribing responsibility to individual politicians for taking Ireland and Apple to task (Kron, 2025). Certain interviewees observed the political system (of many states individually and collectively) as prodding the legal system into dealing with what was observed as a social problem, namely aggressive tax avoidance by multinational enterprises and tax competition by states. Yet, the focus of the research is how the legal system communicated the legality/illegality of the tax relationship between Ireland and Apple.

Thirdly, society is viewed as composed of operatively closed systems cognitively open to environmental influences from other systems. These environmental influences irritate, or prompt, the system into communicating but that communication is confined by the possibilities of that functional system. Again, the legal system was observed as having been nudged, or irritated (Harste and Febbrajo, 2016), into noticing a political event and responding to it, but within the inimitable style and constraints of the legal system. The interviews observed this irritation but also observed what was ignored by the legal system: an overt political context, a lack of explanation as to the delay in taking a state aid action, an emphasis on equitable distribution of the tax burden. In drawing these distinctions, the interviewees positioned the legal system as acutely responsive to environmental pressures while noting, at the same time, that the legal system itself did not mention any such external influences. This distinction was observed as the legal system being able only to communicate legality/illegality: it does not use the semantics of fairness, political compromise or new regulatory standards: it uses the difficult, ambiguous semantics of state aid. This observation of observation rendered visible what the legal communications do not address: the finding of illegality is contingent, even improbable, and replete with unarticulated distinctions between fair/unfair, ethical/unethical.

Focusing on one system is theoretically justified as functionally differentiated communications do not assign a hierarchy to systems (Přibáň and Nelken, 2001). The center is absent, and Luhmann rejects the view that the political system can exert measurable authority over other systems (King and Thornhill, 2003). Instead, each system grows in complexity, its self-referential nature intensifies. This self-reference “unfolds into a linear infinity of never-ending operations of the same system” (Luhmann, 2004, p. 211). There is no “supreme normative framework unifying and integrating contemporary societies” (Přibáň and Nelken, 2001, p. 5) and the development of specifically legal communications secures the place of law in a complex and contingent environment.

There is no immutable system dictating the acceptance of events by other systems; there is only the self-organization or autopoiesis of the system and the reception of its communications in other systems. While systems are accepted as real (Moeller, 2013; Rasch, 2000), communication constructs the reality it pre-supposes in the very act of pre-supposing it: the reality of the relationship between taxpayer (Apple) and tax collector (Ireland) is re-imagined by the legal system in 2013 when the EU first challenged it as prohibited state aid. This re-imagining is observed in the interviews. The novel, yet sustainable, legal approach is debated, thereby supporting the autopoiesis of the system. The distinction in approach between the legal system and the political system is also observed, interpreting the dry semantics of the legal system as both a systemic characteristic but also disguising values of fairness, morality and a legal answer to unethical behavior by both states and multinational enterprises.

The constructivist epistemology chimes with the artificiality of tax. Tax is a creature of language and therefore ambiguous and imprecise (Freedman, 2007; Picciotto, 2015). Tax is constructed by the legal system under direction from the political system (Snape, 2011). It reflects a philosophical stance (O’Neill and Orr, 2018), is informed by political science (Rixen, 2008), and is expressed in sociology (Martin et al., 2009), regulation (Braithwaite, 2007), or corporate social responsibility (Gribnau, 2015). Tax is a legal document, based on legislation and judicial interpretation (Weisbach and Plesko, 2007) but also relevant in accounting (Hanlon and Heitzman, 2010) and economics (Riedel, 2018). Tax law is both complex (Clemens, 1999) and wholly artificial (Freedman, 2007) and displays neither justice nor reason (Prebble, 1994).

Neither taxation nor EU law is known in the physical world; each is the product of a legislative process approximating language to economic and social activity, mediated through communications of the legal system which recursively interpret the application of rules to event. This instability is well-known, and inescapable in systems (Luhmann, 2012, 2013). Laws are open to interpretation, sensitive to contextualization, and applicable to a literally unknowable number of contingencies. The epistemological position of this research is that there are no facts about the legal system and its environment which are not socially produced. An empirical methodology based on systems theory provides one point of observation and the construction of a reality.

All this is a highly abstract rendition of Luhmann’s systems theory, approached through his theory of observation (Andersen, 2003; Rasch and Wolfe, 2000), which is based on distinctions (Luhmann and Rasch, 2002). The challenge for any empirical work based on systems theory is that, despite Luhmann’s assurance that systems are empirically observable in the “real world” (Luhmann and Rasch, 2002, p. 136), the gap between theory and its operationalization is quite vast, and difficult to traverse. Like his mentor, Talcott Parsons, Luhmann stands accused of empirical irrelevance (Vanderstraeten, 2021).

This dearth of empirical work has called into question the status of Luhmann’s grand theory: “Is the lack of an empirical program of research building on Luhmann’s theory fortuitous or does it indicate that this theory should be considered as a philosophy rather than a heuristic for the explanation of operations in social systems?” (Leydesdorff, 2009, p. 150). We now turn to these empirical questions.

## 4 Deciphering the empirical gap

A major issue in empirical research based on systems theory is that the theoretical emphasis on systemic communication crowds out any role for human action or agency. This methodological problem arises from Luhmann’s insistence that sociological analysis cannot limit itself to human interactions (Luhmann, 1995b). System and environment are in a productive relationship: any method of investigating society must reflect this ontological position that autopoietic, or self-organizing, systems communicate (Leydesdorff, 2009). At the same time, there is nothing pre-ordained about a systemic communication: possibilities are abundant and systemic communication is contingent.

There is an ontological fault line running between systems theory and action theory: Giddens (1984) sees systems theory as ignoring the empirical complexity of actors; Honneth (1993) rejects the focus on ‘systemic mechanisms’ over actors. Even Habermas, who sparred with Luhmann and who was influenced by him (Bausch, 1997), sees systemic mechanisms as needing “to be anchored in the lifeworld” (Habermas, 1987, p. 154). The major problem with Habermas’ theory has been the difficulty of reconciling the lifeworld to the system (McCarthy, 1993). This too motivates the type of action-centered criticism that is a recurring feature of dismissive analyses by Luhmann’s critics (Lee, 2000; Wolfe, 1991).

Luhmann self-identified as “radically anti-humanistic” (Luhmann, 2012, p. 12). Luhmann’s systems theory presents a dilemma: pursue the theoretical basis of a scientific study of society, or focus on human action, but suffer from a lack of theoretical sophistication in so doing (Wolfe, 1991). One response, to the criticism of the absence of the individual from the super theory, is advanced by Luhmann himself, where he argues that the theory of autopoietic legal systems takes individuals seriously precisely because a human cannot be part of any system (Luhmann, 1992). This could be viewed as sophistry, an ostensibly smart and simple rejection of social agency. It also leaves unanswered the criticisms of empirical abstraction made in respect of Luhmannian systems theory (and indeed Parsonian social theory) (Vanderstraeten, 2021). Is the “methodological antihumanism” ascribed by Habermas to Luhmann (Rottleuthner, 1989, p. 781) so detached from the individual that any method of operationalization of systems theory which involves interviewing individuals is fated to be theoretically discordant?

When operationalizing systems theory, the physical reality of individuals cannot be ignored. Clearly, society is populated by actual people: individuals from Apple and the Irish state came together to agree tax rulings in 1991 and 2007; the board of directors of the subsidiaries of Apple Inc. were physically located *somewhere* when decisions were made; the Apple intellectual property was developed by engineers located in California; functionaries employed by the EU in Brussels initiated the legal actions against Ireland and Apple (Hunt and Doyle, 2025). Each of these individuals have cognitive processes, and differ in their experiences and motivations.

Yet these individuals can be positioned as having roles, not individual identities: “character-masks” (Teubner, 1989, p. 741). Recognizing individuals, while studying communications, has important consequences for the methodology: it accords with Luhmann’s emphasis on a systems perspective, independent of human agency, but nonetheless facilitates a qualitative methodology, where meaning is constructed through interviews with human agents. These “role-bundles, character-masks” (Teubner, 1989, p. 741) are highlighted during the process of data selection, as each interviewee observes him or herself as having a role (see Table 1).

**Table 1:** List of interviewees and dates interviewed.

Identity of interviewee	Date of interview
European commission employees	
EC1	June 2017
EC2	June 2017
EC3	January 2018
EC4	July 2018
EC5	July 2018
Former Irish officials	
Irish official 1	September 2017
Irish official 2	January and July 2018
US officials	
Former US treasury official	July 2017
Former judges	
Former EU judge	June 2017
Former Irish judge	September 2017
Former Finnish judge	November 2018
Irish tax lawyers (ITL)	
ITL1	May 2017
ITL2	June 2017
Irish state aid lawyers (ISAL)	
ISAL1	August 2017
ISAL2	January 2018
ISAL3	October 2018

Despite an understanding of how wide-ranging and internally consistent Luhmannian systems theory is Jacobson (1989), Lempert (1987), and Sinclair (1992), there is a clear problem with empirical work based on this theory (Campilongo et al., 2020). Systems theory is censured for not being capable of validation through empirical evidence and for the absence of factual evidence of autopoiesis (King and Thornhill, 2003). Baghai (2015) cautions against too facile an integration of systems theory and empirical investigation. The criticism is multidisciplinary. Within organization studies, there is an awareness that Luhmann makes methodological demands that could well discourage more than one student (Hernes and Bakken, 2003). Leydesdorff (2009), in the context of the study of communications, sees the bridge between theory and empirical research as being a crucial step as only theories which can be operationalized can be methodologically informed. Some leads as to empirical application are given by La Cour et al. (2007), albeit at the same time as they decry the lack of empirical application of Luhmann’s work. On the other hand, Knudsen (2010) sees the functional method as enabling fruitful empirical work. Besio and Pronzini (2008) view empirical work as possible, so long as the epistemological implications of systems theory are recognized. This is a key point: the form of observation must respect the contingency of observation, and the focus on the system, and its structural possibilities, must be maintained (Staller and Koerner, 2025).

Charges of abstraction and empirical emptiness (Black, 2000; Cotterrell, 1992; Münch, 1987; Rottleuthner, 1989) nonetheless remain. They may even explain the relative sparseness [particularly in the Anglosphere (Jokisch, 2011)] of empirical research using Luhmannian systems theory as an epistemological framework. The lack of empirical application of Luhmann’s systems theory may explain why it functions more as a general or meta-theory of society (Moeller, 2013). Paterson and Teubner (1998) proffer a response to these criticisms: the absence of theory is productive of a methodology which, while sophisticated, is based on poor theorizing, whereas theory has become philosophical and speculative, relying on poor empirical support. Lempert puts it pithily when he notes: “Theory is not just theoretical; it is useful” (Lempert, 2010, p. 893). The close integration of theory and method is, therefore, a pre-occupation of this paper.

## 5 Materials and methods

The research received ethical approval for the conduct of interviews from University College Dublin. Interviewees took part on the basis of voluntary, informed written consent to the publication of pseudo-anonymous analysis. The population of interested parties was relatively discrete, as only a certain number of people has sufficient knowledge of the legal system relating to state aid and cross-border taxation, to comment meaningfully on it. This is not a requirement of systems theory, that observers need to be expert, merely a pragmatic attempt to ensure that the interview data was comprised of as many knowledgeable views as possible and that different “character masks” (Teubner, 1989, p. 741) could be ascribed to the interviewees. It is also an acknowledgment that the interviewees possess “the street smarts of the socially experienced participants in social communication” (Luhmann, 2012, p. 14), and treating their utterances as ‘data’ detracts from the main theoretical focus: why do most possibilities not come to fruition and ‘how does society manage to sort out what is possible?’. The state aid action by the European Commission was never presented as the inexorable application of law to circumstance, but as a contingent event thrown up by a confluence of environmental pressures (Luhmann, 1995a) and subjected to the specific, autopoietic process of the legal system to be ruled illegal (Teubner, 1989).

Potential interviewees were differentiated according to their institutional identity: Ireland, Apple and the European Commission. Only the last category was largely receptive to an invitation to interview (although some European Commission officials declined to be interviewed). Interviews with two individuals who identified as institutionally connected with Ireland were carried out. Emails and telephone calls seeking to recruit interviewees institutionally connected to Apple went unanswered. The Irish Officials, the US Treasury Official and the judges were all former employees of the relevant organization. A decision was taken to balance observations from the European Commission officials with interviews of Irish lawyers and former judges who were sufficiently familiar with tax and state aid to likely to provide an informed observation. The absence of interviewees who self-identify as Apple employees is accepted as being an unmarked space from which there are no observations (Rasch and Wolfe, 2000). It is a known blind spot.

The interviewees were selected according to criteria of knowledge of the subject matter of state aid and/or tax, together with network sampling, i.e., each interviewee was asked to suggest a further suitable interviewee (Roulston and Choi, 2018). First, the officials of the European Commission were identified through an internet search of relevant news stories about the Apple case. This gave a short-list of potential interviewees. Secondly, lawyers working in either taxation or state aid were identified using a search of specialized legal media and a search of the websites of major law firms. Overall, 26 requests for interview were issued, and 16 interviews were completed between May 2017 and November 2018. These details are set out in Table 1.

The interviewees were labelled, in accordance with their “character-masks” (Teubner, 1989, p. 741), with their dominant, de-personalized, self-description. This is a self-description recognized, even generated, by the legal system: lawyer, official, judge. The interviewees were asked to describe their role in the organization and their area of expertise. Where there was more than one interviewee within the same classification (e.g., European Commission officials), the interviews were numbered in accordance with the date of interview (e.g., EC1, EC2 etc.).

The interview guide loosely followed during the semi-structured interviews addressed whether economic or political concerns prompted legal action, or whether the legal system is autonomous in both directing its attention to circumstances and finding those circumstances to be illegal. The semi-structured interviews asked whether the interviewees had previously seen state aid laws as a risk factor in tax planning for multinational companies and asked whether the action by the European Commission was surprising (a discussion closely related to the strong possibility of the legal system never communicating on this issue (Luhmann, 2012)). The interviewees were therefore asked how the European Commission came to act in 2013, and not before, or never. Interviewees observed that there were problems in international taxation. These problems ranged far: tax competition by states leading to a “race to the bottom” in international tax; power of multinational companies to dictate an economic agenda; aggressive tax planning by multinational companies; lack of political will to harmonize EU tax policy. Wide discussion of these problems had taken place in the US Senate, the UK Parliament and in the media (Hunt, 2020) and the interviewees referred to this political and media coverage. Interviewees often added expressions of disapproval, or explanation for the tax practices of Ireland and Apple: lack of fairness, immoral or indecent tax practices by states and multinational companies; the benefits of certainty of tax liability. The legal action was described as a response to political pressure, an effort to demand accountability from Ireland, and Apple, for their mutually beneficial tax relationship which, the European Commission argued, was illegal.

The purpose of the semi-structured interviews was to generate second-order observation, external to the legal system, on the communications of the legal system relating to the European Commission’s investigation of Ireland. The mechanics of analyzing the interview data involved a thematic analysis (Braun and Clarke, 2006), using NVivo as an analytical aide. But the most important part of the analysis was to maintain an awareness of the epistemological basis of systems theory: there is no single means of determining the world “as it is” (Teubner, 1989); there is just the “illusion of reality” (Luhmann and Rasch, 2002 p. 50). This is a recognition of modern society as “a polycentric, polycontextual system” (Luhmann and Rasch, 2002 p. 52) which frames reality according to the distinctions observed by the system.

This is a difficult theoretical framework to operationalize (Andersen, 2003, 2011) through the generation of interview data – which of necessity involves human participation – but also through the analytical strategy. The purity of the focus on observation, rather than consciousness, finds expression through excising questions relating to feelings and emotions (Rennison, 2007) but, even more importantly, ensuring that the analysis is based on the communication of the interviewee, expressed through language. Language is defined as the “medium that increases the understandability of communication beyond the sphere of perception” (Luhmann, 1995b, p. 160). Language is not a sign system; its true function “lies in generalizing meaning with the help of symbols” (Luhmann, 1995b, p. 94). This is not to say that gestures, and implicit presentation of viewpoints are impossible, even in Luhmannian terms, to communicate, but merely to accept that clearer adherence to Luhmann’s theory of a communicative society is facilitated through straightforward analysis of what is communicated through language. The communication of meaning through language leaves traces in the empirical world, and the analytical strategy was designed to search for those traces in language, not the interviewer’s impressions or feelings. The analytical strategy respected the epistemological basis of systems theory through an awareness of the sheer unlikeliness or contingency (Luhmann, 1995b; Zolo, 1986) of a legal case taken by the European Commission in 2013 in respect of a tax relationship which had been in place since 1991 or, on a timeline relevant outside the legal system, since 1980.

This analytical strategy comprised a functional analysis, viewing the state aid action as a solution to problems. A functional analysis of the system views the solution as indissoluble from the problem (Knudsen, 2010; Hanna, 2017) but also recognizes contingency as there is no single, ready-made solution to the problem (Boyden, 2011). While the problems outlined above were articulated by observers, the legal system communicated only in the technical language of state aid, without explicit recourse to wider social problems or political values. The autopoietic legal system could only bring forth a finding of illegality based on highly technical state aid rules. The legal system was observed as using an impermeable terminology – objective, passive, measured and complex – to describe Ireland and Apple’s tax relationship as illegal.

This was support for functional differentiation, where the system is separate from its environment (Luhmann, 2012). The singularity of the language of the legal system, and the silence about any political or moral views on the fairness of Ireland and Apple’s tax practices, sustain the autopoiesis of the legal system and its separateness.

The second part of the analytical strategy was to observe the values (Von Groddeck, 2011; Hunt and Doyle, 2025) attributed to the legal system, or prodding it to action. This was juxtaposed with the silence of the legal system about morality, or fairness: the legal system was deeply grounded in discussions about technical aspects of state aid, selectivity, reference frameworks and time limits (Mason and Daly, 2024). The interviewees had also placed the legal action within a particular political context: discussions about Apple and other multinational companies in the US Senate and the UK Parliament. This sustained an interpretation of the legal system being observed as having been irritated into an investigation of Ireland and Apple. This structural coupling was theoretically explained as being an environmental irritation of the legal system by the political system (Harste and Febbrajo, 2016). The final point of observation was then to assess these environmental pressures – irritations – and seek to understand how the legal system communicated a solution at a particular moment in time, 2013, and not some other time or not at all.

Environmental pressures were observed: the legal system was seen as having been irritated into action by political and media discussion in the USA and the UK (Hunt, 2020; Hunt and Doyle, 2025). In distinction to the ostensibly valueless communications of the legal system, the external perspective on the Ireland-Apple tax rulings was replete with moral and value observations. These observations were placed outside the boundary of the legal system; the problems to which the legal system was observed as providing a solution – or a vindication of the values of fairness and morality – were not themselves articulated by the legal system.

The detailed analysis of the interviews, together with the doctrinal analysis of the legal argumentation in the Ireland-Apple case are dealt with elsewhere (Hunt and Doyle, 2025; Hunt, 2025). This paper is concerned only with the methodological paradox, within Luhmannian systems theory, of drawing upon the observations of elite individuals to construct the meaning and impact of a communication of the legal system.

## 6 Discussion: coherence and limitations of empirical application of systems theory

Luhmann doubts that there is any correlation between empirical research and social reality, except through the methodologically unguided “active interpretation” of results (Luhmann, 1992, p. 1438). This is a provocative statement, which no doubt underlies certain criticisms of Luhmann as empirically ineffective (Rottleuthner, 1989) or empirically irrefutable (Münch, 1992). Luhmann’s suspicion of empirical methods is theoretically explicable: while systems theory accepts the existence of the system and its environment, it views reality as contingent and socially constructed (Luhmann, 1995b). Thus an empirical method designed to discover *the* truth or reality of a particular event or phenomenon is doomed to failure (King and Thornhill, 2003). That is simply not what systems theory considers possible. The analysis of the interviews is one reality, amongst many possibilities, of how the legal system came to communicate, from 2013 to 2024, on the tax relationship between Ireland and Apple.

Furthermore, empirical methods are not sufficiently developed to verify theoretical positions: Luhmann refers to the “uncomfortable tension between theoretical conceptions and the present possibilities of empirical research” (Luhmann, 1992, p. 1439). This informs the view that what empirical research produces is no closer to a real view of society than theory; empirical research produces “highly artificial constructs, excessively selective abstractions, mere internal artefacts of the scientific discourse that are both as real and as fictional as are theoretical constructs” (Paterson and Teubner, 1998, p. 455). Teubner (1989) views an autopoietic legal system as implying, even requiring, an abandoning of the naïve reality assumption that human actors are the basic elements of society.

Yet systems theory is useful precisely because it accepts the limits of empirical methods, and sees them in a theoretically informed manner as effecting a search for possibilities (Andersen, 2003) and the potential for interpreting reality from a different point of observation (King and Thornhill, 2003). It also views autopoietic systems, functionally differentiated and separate from their environment, as constraining possibilities. Given the artificiality of tax (Freedman, 2007) and its reliance on legislative language (Picciotto, 2015) as constraints on action, the tax system is ontologically and methodologically prior (Dawe, 1970) to its numerous participants. The legal system both enabled challenge of Ireland and Apple’s tax relationship but, at the same time, constrained the grounds for this challenge. Any argument against Ireland had to be fitted into the state aid straitjacket.

There are several points which can be made on the methodology. Principally, foregrounding systemic constraints has the effect of moving away from an individualistic methodology, where agents are questioned about who the prime mover was, what was their motivation and who stood in the way of action (Luhmann, 2012). The empirical approach based on Luhmann’s system theory translates sociological inquiry “from the form of ‘what’ to the form of ‘how’” (Luhmann, 2004, p. 451). This is an important re-orientation, as the question becomes one of how autopoiesis of the system is maintained and how the legal system operates under the impact of permanent irritation by the environment.

The de-personification of individuals might just be seen as a device for hiding individual agency. Operationally, people undertake specific actions and make particular choices (Mingers, 2002). While systems theory has a theoretical purity, Mingers see this as having been obtained at the cost of “an incredibly abstract and reductive view of the social world” (2002, p. 292). Nobles and Schiff (2020), when discussing complexity theory, write that autopoietic systems theory rejects “the suggestion that society could function on the basis of the aggregation of the subjectivities of more than 7.5 billion subjects” (Nobles and Schiff, 2020, p. 672). Nobles and Schiff use an apt metaphor when supporting the examination of court judgments rather than judges: “this is to mistake the persons moving the lever for the lever to be pulled” (Nobles and Schiff, 2017, p. 105). The role of the individual and the existence of social structures is an ongoing debate, but subscribing to systemic autopoiesis requires some acceptance of the existence and efficacy of social structures (Mingers, 2004).

The shift to second-order observation is a consequence of the functional differentiation of an increasingly complex modern society. Interviewing experts, rather than random individuals, is a research choice designed to elicit a knowledgeable insight into how the legal system reached the view it did, and how its communications are both prompted and interpreted in the environment. Although there was a focus on choosing individuals involved in the triangular dispute, this is compatible with acknowledging that a different construction could emerge from other points of observation. In all this, the individual is seen as outside all systems, outside a society composed of communications. The antihumanism is off-putting, it seems disrespectful to state that society is “indifferent to what happens in the consciousness of the individual” (Luhmann, 2013, p. 102). This indifference is, on its face, rather dismaying. Yet it is based on an understanding of the individual being at the mercy of structural constraints and institutional power (Philippopoulos-Mihalopoulos, 2009). The orientation of social inquiry away from a focus on a (small number) of individual agents serves to highlight systemic possibilities (and impossibilities). Focusing on systems – asking how, not who – means that there is no distraction from inquiring into structural and social constraints.

Nonetheless, this research is cognizant that an entirely different viewpoint, based on questions of human agency – an analysis of how individual agents craft the Ireland-Apple tax relationship – could inform the research. There is a good deal of research on tax avoidance and state competition from, broadly, an agency perspective (Addison and Mueller, 2015; Mulligan and Oats, 2013; Radcliffe et al., 2018), and even from the point of view of state agency (Sharman, 2007; Webb, 2004). Treating individual activity and normative values as the yardstick for an assessment of a tax relationship enables a finding of sub-optimal agency. This is useful but it ignores any discussion of the functional or systemic characteristics of tax, or the constraining influences of the legal, economic or political system on taxation. In short, this is no more than an example of a sociological dichotomy: “a sociology of social system and a sociology of social action” (Dawe, 1970, p. 214), representing a conflict between a view of systems as socially constraining and a view of action as empowering individual agency (Van Krieken, 2002).

The failures of empirical work based on systems theory are not fatal because that failure is theoretically explicable. Empirical work informed by systems theory is aimed at understanding a chaotic, complex and competing cacophony of social communication. In the chaos of modernity, the possibilities of non-hierarchical systems are based on an awareness of contingency: things could always be other (Luhmann, 1995b). The political system cannot be relied upon to formulate a response to all problems: it is not pre-eminent (King and Thornhill, 2003). Social order is not based on discerning the motivations or cognitive processes of individuals but on functionally differentiated systems seeking to stabilize expectations within that function (Luhmann, 2012). In a time of increased social upheaval and unrest, the importance of focusing on systems and structures rather the individual facilitates a less distracted, more measured approach. This is a modest, but necessary, endeavor.

## Data Availability

The raw data supporting the conclusions of this article will be made available by the authors, without undue reservation.
